# Genetic Variability of the Neogregarine *Apicystis bombi*, an Etiological Agent of an Emergent Bumblebee Disease

**DOI:** 10.1371/journal.pone.0081475

**Published:** 2013-12-06

**Authors:** Jafar Maharramov, Ivan Meeus, Kevin Maebe, Marina Arbetman, Carolina Morales, Peter Graystock, William O. H. Hughes, Santiago Plischuk, Carlos E. Lange, Dirk C. de Graaf, Nelson Zapata, Jose Javier Perez de la Rosa, Tomás E. Murray, Mark J. F. Brown, Guy Smagghe

**Affiliations:** 1 Department of Crop Protection, Faculty of Bioscience Engineering, Ghent University, Ghent, Belgium; 2 Laboratorio Ecotono, Centro Regional Universitario Bariloche, Universidad Nacional del Comahue, Instituto de Investigaciones en Biodiversidad y Medioambiente, Consejo Nacional de Investigaciones Científicas y Técnicas, Bariloche, Río Negro, Argentina; 3 Universidad Nacional de Río Negro, Sede Andina, Bariloche, Argentina; 4 School of Biology, University of Leeds, Leeds, United Kingdom; 5 School of Life Sciences, University of Sussex, Brighton, United Kingdom; 6 Centro de Estudios Parasitológicos y de Vectores, Centro Científico Tecnológico La Plata, Consejo Nacional de Investigaciones Científicas y Técnicas, and Comisión de Investigaciones Científicas de la provincia de Buenos Aires, La Plata, Argentina; 7 Department of Physiology, Faculty of Sciences, Ghent University, Ghent, Belgium; 8 Departamento de Producción Vegetal, Facultad de Agronomía, Universidad de Concepción, Chillán, Chile; 9 Centro Nacional de Servicios de Constatacion en Salud Animal, Jiutepec, Morelos, Mexico; 10 Department of Zoology, Institute for Biology, Martin-Luther University Halle-Wittenberg, Halle, Germany; 11 School of Biological Sciences, Royal Holloway, University of London, Egham, United Kingdom; CNRS, France

## Abstract

The worldwide spread of diseases is considered a major threat to biodiversity and a possible driver of the decline of pollinator populations, particularly when novel species or strains of parasites emerge. Previous studies have suggested that populations of introduced European honeybee (*Apis mellifera*) and bumblebee species (*Bombus terrestris* and *Bombus ruderatus*) in Argentina share the neogregarine parasite *Apicystis bombi* with the native bumblebee (*Bombus dahlbomii)*. In this study we investigated whether *A. bombi* is acting as an emergent parasite in the non-native populations. Specifically, we asked whether *A. bombi*, recently identified in Argentina, was introduced by European, non-native bees. Using ITS1 and ITS2 to assess the parasite’s intraspecific genetic variation in bees from Argentina and Europe, we found a largely unstructured parasite population, with only 15% of the genetic variation being explained by geographic location. The most abundant haplotype in Argentina (found in all 9 specimens of non-native species) was identical to the most abundant haplotype in Europe (found in 6 out of 8 specimens). Similarly, there was no evidence of structuring by host species, with this factor explaining only 17% of the genetic variation. Interestingly, parasites in native *Bombus ephippiatus* from Mexico were genetically distant from the Argentine and European samples, suggesting that sufficient variability does exist in the ITS region to identify continent-level genetic structure in the parasite. Thus, the data suggest that *A. bombi* from Argentina and Europe share a common, relatively recent origin. Although our data did not provide information on the direction of transfer, the absence of genetic structure across space and host species suggests that *A. bombi* may be acting as an emergent infectious disease across bee taxa and continents.

## Introduction

Bumblebees (*Bombus* spp.) are essential generalist pollinators [1], but many of their populations are undergoing major losses. While range decline for some species has been moderate, others are vanishing rapidly [2], [3]. Although the trend of decline is evident worldwide, the responsible drivers are diverse (for review see [4]), with each driver potentially interacting with other drivers and acting differently across geographic locations [5]. This complexity makes it difficult to clarify the response to a single driver.

The relatively unspoiled temperate forests of southern Argentina and Chile are the natural habitat of *Bombus dahlbomii*, the largest bumblebee species in the world [6], [7,]. Populations of *B. dahlbomii* appear to be in a steep decline, which has coincided with the recent establishment of the non-native European bumblebee, *Bombus ruderatus,* and more recently of *Bombus terrestris* [8], [9]. *Bombus ruderatus* was intentionally introduced into southern Chile in 1982–1983 from a New Zealand population, which was also an introduced population originating from the UK [10], and has subsequently migrated from Chile into Argentina [6], [7]. *Bombus terrestris* became established in Chile after the introduction of commercially produced colonies from Belgium and Israel for crop pollination around 1998 [9]. It has been hypothesized that the decline of *B. dahlbomii* may be partly due to pathogen spillover from introduced bees [11], and in particular that the introduced bumblebee *B. terrestris*, may be the carrier of novel pathogens into the environment.

Commercially produced bumblebee colonies have been shown by many studies to carry a wide range of microbial parasites [12], [13], [14], [15], and such an introduction of new pathogens could induce emergent infectious diseases with dramatic consequences for native populations [14]. *Apicystis bombi*, a neogregarine parasite (Apicomplexa: Neogregarinorida), has been found to be present in Argentina infesting in native and non-native bumblebees, as well as the honeybee *Apis mellifera* [11], [16], [17], and thus it may be an important pathogen driving emerging infectious disease, and potentially involved in the decline of *B. dahlbomii* in this region. However, pathogenicity of *A. bombi* in *B. dahlbomii* needs to be determined in order to infer the risk associated with its spillover.

*A. bombi* was first discovered in Italy in 1988 and has now been recorded in nearly 20 *Bombus* species [18], [19] including in commercially produced bumblebee colonies [13], [15]. Although empirical data on the pathology of the parasite are limited, the fat body of infected bumblebees is destroyed due to the proliferation of the pathogen [20], and its presence correlates with high mortality in infected spring queens, preventing them from establishing colonies [21], [22]. Consequently, upon entering novel host populations, *A. bombi* may have the potential to act as an emergent infectious disease. However, to date it is unknown whether the *A. bombi* present in the populations of European bumblebees and honeybees established in Argentina, was acquired *in situ* or if it was co-introduced from Europe with them. A previous study using microscopy found *A. bombi* in the invasive *B. terrestris* in northwest Patagonia, but not in native bumblebees from regions which are currently free of *B. terrestris* [16]. Similarly, using molecular techniques, *A. bombi*-infected honeybees were only observed in regions invaded by *B. terrestris*, while the pathogen was not detected in honeybees from *B. terrestris*-free regions [17]. However, *B. terrestris-*free regions were also geographically and climatologically different from the regions where *A. bombi* was found and, in the absence of epidemiological knowledge, these observations cannot by themselves definitively prove that *A. bombi* infections resulted from the introduction of *B. terrestris*. More recently, Arbetman et al. (2013) found that 14 out 30 *B. terrestris*, five out nine *B. ruderatus* and one out of nine *B. dahlbomii* specimens collected in northwest Patagonia after invasion by *B. terrestris*, were infected with the neogregarine *A. bombi* [11]. Conversely, the pathogen could not be detected in any of the 30 of *B. ruderatus* and 52 *B. dahlbomii* museum specimens, collected before the invasion of *B. terrestris* [11]. However, this too is not definitive, because detection limits in the ethanol-stored samples [11] and thus the original source of this parasite in South America is still controversial. Finally, recent molecular screening of honeybee hives (n = 363) in Europe (Belgium) found 40.8% to be positive for *A. bombi* [23] while in Japan two of 69 examined hives (2.9%) were positive [24]. However, whether the *A. bombi* discovered in honeybees are of the same strain as those found in *Bombus* species, and whether interspecific transmission is possible, remains unknown.

In order to study the interspecific variability of *A. bombi* present in non-native bees that are established in Argentina, i.e. *B. terrestris*, *B. ruderatus* and *Ap. mellifera*, we sequenced the highly variable internal transcribed spacer 1 (ITS1) and ITS2 regions as genetic markers. We focused on two questions: (i) do honeybees and bumblebees share the same haplotypes with *A. bombi*, and (ii) do parasite strain haplotypes found in Argentina and Europe exhibit geographical structure? Answering these will provide insight into the transmission dynamics and native range of *A. bombi*.

## Materials and Methods

### Origin of Infected *A. bombi* Species

**Figure 1:**
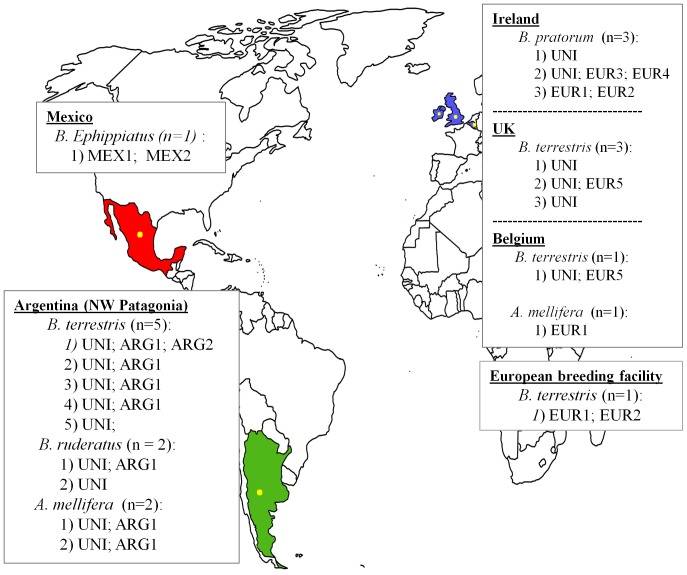
Bee sampling infected with *Apicystis bombi* in different geographical areas. Overview of bumblebee (*Bombus pratorum*, *Bombus terrestris*, *Bombus ephippiatis* and *Bombus ruderatus*) and honeybee (*Apis mellifera*) samples included in the study, including the numbers of bees from each species at each location. All bees were infected with *Apicystis bombi* and the haplotypes found in each bee are given (UNI, EUR1-5, ARG1, ARG2, MEX1 and MEX2).

To determine the haplotypes of *A. bombi* present in Argentina, we collected nine *A. bombi*-positive specimens: *B. terrestris* (n = 5), *B. ruderatus* (n = 2) and *Ap. mellifera* (n = 2). All samples were collected in northwest Patagonia [11], [17]. The European sampling consisted of eight *A. bombi*-infected specimens: *Bombus pratorum* from Ireland (n = 3; [22], *B. terrestris* from Belgium (n = 1) and the UK (n = 3), and *Ap. mellifera* from Belgium (n = 1). Two extra specimens were analyzed: one *B. terrestris* from a commercially produced colony in Europe [15], and one *B. ephippiatus* native from Mexico (**Figure 1**). All samples from Argentina were ethanol-stored [11], [17], and the other samples were stored at −20°C before extraction. All samples were extracted with EZNA® Insect DNA kit (Omega Bio-Tek; Norcross, GA) [25] with the exception of the samples from the UK which were extracted with a Chelex extraction protocol.

### Ethics Statement

No national permissions were required to collect samples from the public lands in the locations of Belgium and the UK. Specific permission granted for sampling by the National Botanic Gardens, Ireland, and The Royal Parks, UK. Permits were not required in Argentina for the collection of exotic invertebrates (*B. terrestris,* and *Ap. mellifera*) from public lands. Permission to collect samples on his private land was provided by Luis Rovera in Argentina. Permission to collect bumblebees (*B. terrestris* and *B. ruderatus*) from Argentine National Parks were obtained from Administracion de Parques Nacionales (Argentina), while permission to export them from Argentina were granted by Secretaria de Desarrollo Sustentable y Medio Ambiente. The field studies did not involve endangered or protected species.

### Haplotyping of *A. bombi*

In order to determine the haplotypes of *A. bombi,* we sequenced both ITS1 and ITS2, which are both highly variable regions of the rRNA. Species-specific primers were designed: ApiITS732F 5′-TGGAAACAAGTCATTTTTGGAA-3′ and ApiITS732R 5′-CCTGTTCACTCGCCGTTACT-3′, amplifying an approximately 730 bp long fragment of *A. bombi* within a bumblebee DNA extract. The amplicon contained ITS1, 5.8S rDNA, and ITS2. PCR reactions were done in 25 µl-reaction volumes, containing 0.2 mM dNTPs, 0.5 µM primers, 1×PCR buffer, 1.5 mM MgCl_2_, 1.25 units of Taq polymerase (Taq DNA Polymerase, recombinant/Invitrogen) and 1 µl DNA template. Amplification was performed using one cycle of 94°C for 3 min, followed by 30 cycles of 95°C for 30 s, 55°C for 30 s, and 72°C for 60 s, and a final elongation step of 3 minutes.

PCR products were cloned using the Sticky-End Cloning Protocol (pJET1.2/blunt Cloning Vector, Fermentas UAB, subsidiary of Thermo Fisher Scientific Inc. V. Graiciuno 8 LT-02241, Vilnius, Lithuania). The plasmid was purified with the E.Z.N.A. Plasmid Mini Kit (Omega Bio-Tek) from 10 bacterial colonies containing the ITS regions, and sequenced (LGC Genomics GmbH (Germany, Berlin). In order to analyze the different haplotypes, the sequences were aligned using the BioEdit sequence alignment editor. Not all mutations can be regarded as a different haplotype. It is known that PCR artifacts can occur, with a PCR error rate of ε = 0.555×10^−3^ (1.85×10^−5^ miss incorporations per cycle for 30 cycles) and the probability that a single mutation is caused by PCR error for an amplicon with size 730 bp is *P* = 0.063. This calculation is based on the binomial probability mass function reported by [26,]. To be conservative, we decided to exclude all mutations which occurred only once in the total dataset. Of the remaining mutations, all haplotypes were confirmed at least three independent times. This procedure resulted in eight different haplotypes in our total data set (n = 18). The exclusion of possible PCR errors could lead toward an under-estimate of the true diversity present. Hence PCR products harboring 3 unique mutations (*P* = 0.0008) were subsequently re-included in the dataset. In this way, we incorporated two additional haplotypes, both found within the single Mexican sample. The ITS1 contained three mononucleotide repeats, one of 10 nucleotides in length and two of more than 10 nucleotides. It is known that these regions are, because of polymerase slippage, sensitive to mutation errors [27]. All sequences deposited at GenBank contain the consensus amount of nucleotides plus an N to indicate the unknown amount of extra nucleotides.

### Genetic Variation and Structuring of *A. bombi*

The pairwise mutational differences between haplotypes was calculated. In order to reveal population structuring, the genetic variation within versus among populations was determined using an analysis of molecular variance (AMOVA; Arlequin v3.1). Differences in the average within-population sequence divergence of haplotypes of Europe and Argentina were tested using a Mann-Whitney U test. To reconstruct the phylogenetic tree and estimate evolutionary relationships of the haplotypes, the Neighbor-Joining method was used [28]. Evolutionary analyses were conducted in MEGA5 [29]. Reduced Median (RM) networks were drawn to visualize the evolutionary relationship between different haplotypes using NETWORK 4.6.1 [30]. The Median Joining (MJ) algorithm resulted in the same topology.

## Results

### *Bombus spp.* and *Ap. mellifera* are Infected with the Same Species of *Apicystis*

We collected neogregarine infected bees (specimens) from three different locations: Argentina, Europe and Mexico. After sequencing analysis of a part of the 18S region we confirmed that all hosts were infected with *A. bombi*. All were identical to previous samples of *A. bombi* found in Argentine *B. terrestris*, *B. ruderatus, B. dahlbomii* [11] and *Ap. mellifera* [17], but also to *A. bombi* found in *B. pratorum* in Europe [25]. One mutation in the 18S was observed when comparing it with the 18S fragment found in the native Mexican bumblebee *B. ephippiatus* (KC951279).

With the use of *A. bombi* specific primers, located in the 18S rRNA and 26S rRNA, we were able to sequence ITS1 and ITS2 (GenBank KF322207–KF322216). From each specimen (n = 18) we sequenced 10 different clones; in total seven bees contained one unique haplotype, nine contained two haplotypes and only two bees contained 3 haplotypes. Most specimens (16 out of 18) therefore contained only one or two different haplotypes, indicating that there is little or no intragenomic variability present in *A. bombi*. When analyzing the molecular variance of the complete data set, 34% of the variability in the data was explained by variation within one specimen (**Table 1a**).

**Table 1:** Analysis of molecular variance (AMOVA) showing the distribution of genetic variation in *A. bombi* across (a) all specimens (grouped by location), (b) specimens from Argentina (grouped by host), and (c) specimens from Argentina and Europe (grouped by location).

Source of variation	DF	Sum ofsquares	Variancecomponents	Percentageof variance	*P*	
a) Among regions (Argentina; Europe and Mexico)	2	13.8	0.15	31.8	>0.0000	
Within each region						
Among specimens within each region	15	23.4	0.16	33.8	>0.0000	
Within specimens	138	21.8	0.16	34.4	>0.0000	
b) Among hosts	2	2.6	0.04	17.3	0.06	0.17Fct
Within each host						
Among specimens within each host	6	1.9	0.01	5.5	0.15	0.07Fsc
Within specimens	2	13.4	0.2	77.2	0.005	0.22Fst
c) Among regions (Argentina and Europe)	1	5.8	0.06	15.2	0.0004	0.15Fct
Within each region						
Among specimens within each region	15	23.4	0.16	42.9	>0.0000	0.51Fsc
Within specimens	137	20.8	0.15	41.9	>0.0000	0.58Fst

*P* is the probability of having a more extreme variance component and F-statistic than the observed values by chance alone. F-statistics is a measure for genetic variation with Fct, Fsc and Fst assessing different hierarchical levels of subdivision.

To study *A. bombi* transmission among different host species, we investigated the Argentine subsample (n = 9). Here three different species were sampled in close proximity to each other (samples were at most 25 km apart). We determined whether haplotypes were distributed randomly among these three different species (*B. terrestris,* n = 5; *B. ruderatus,* n = 2; and *Ap. mellifera*, n = 2). The two most common haplotypes were present in all three host species (**Figure 1** **,** **2**). The most common haplotype (UNI) was detected in all nine Argentine bees, while the other prevalent haplotype (ARG1) was also found in seven of these specimens (**Figure 1** **,** **2**). Only 17.3% of the total genetic diversity of *A. bombi* was explained by differences among host species. It should be noted though, that this percentage is a consequence of the higher sampling of *B. terrestris* resulting in a unique haplotype (ARG2) for this species, and the majority of the genetic diversity was found within host species: 5.5% among specimens of one host species and 77.2% within a specimen (**Table 1b**).

**Figure 2:**
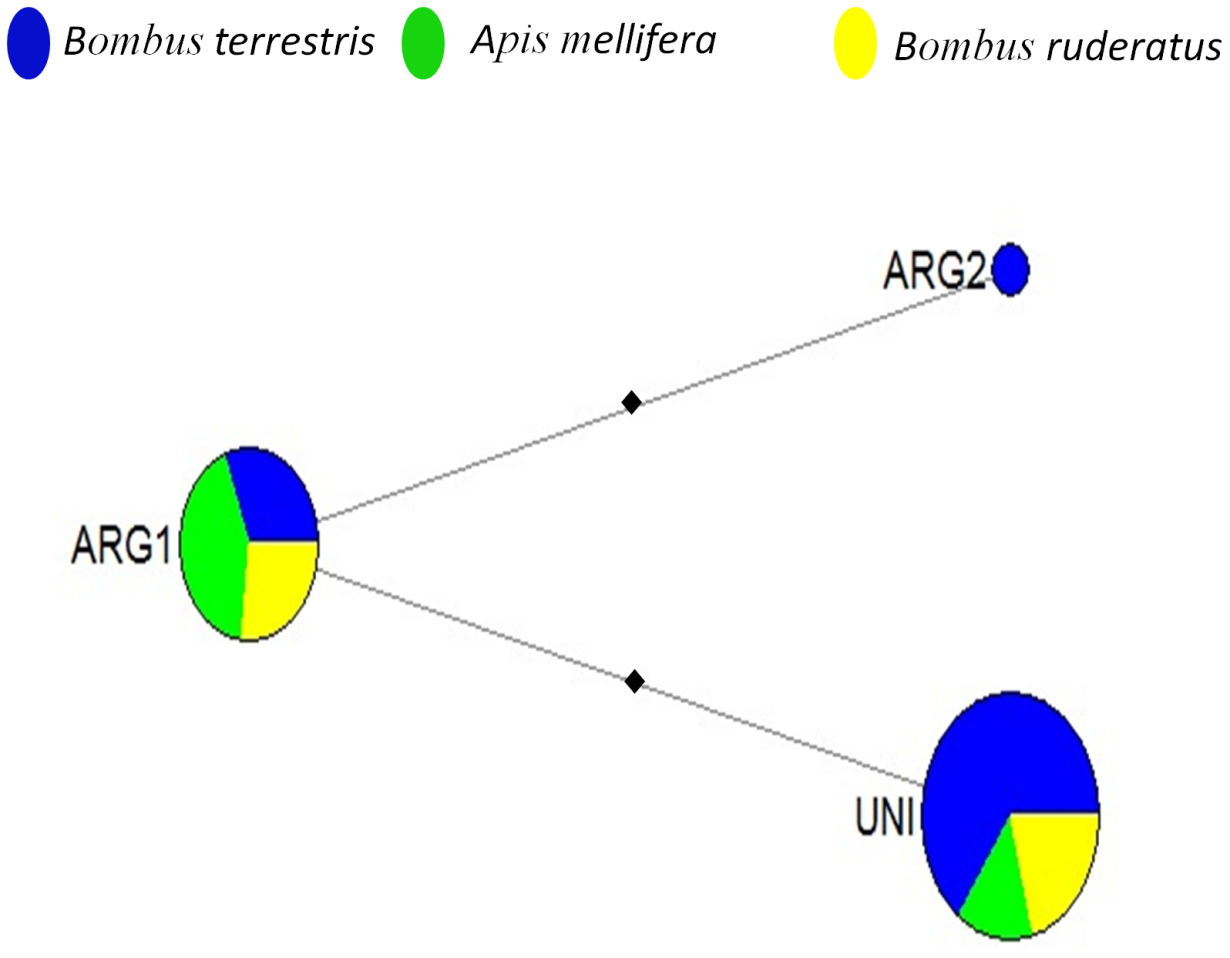
*Apicystis bombi* haplotype abundance in three introduced bee species in Argentina. The size of the node represents the relative abundance of a haplotype and the different colors indicate the proportion of samples with a haplotype that was present in *Bombus terrestris* (blue), *Bombus ruderatus* (yellow) or *Apis mellifera* (green). The black squares represent unobserved single-nucleotide substitutions.

### Argentine and European *A. bombi* Share the Same Origin

**Figure 3:**
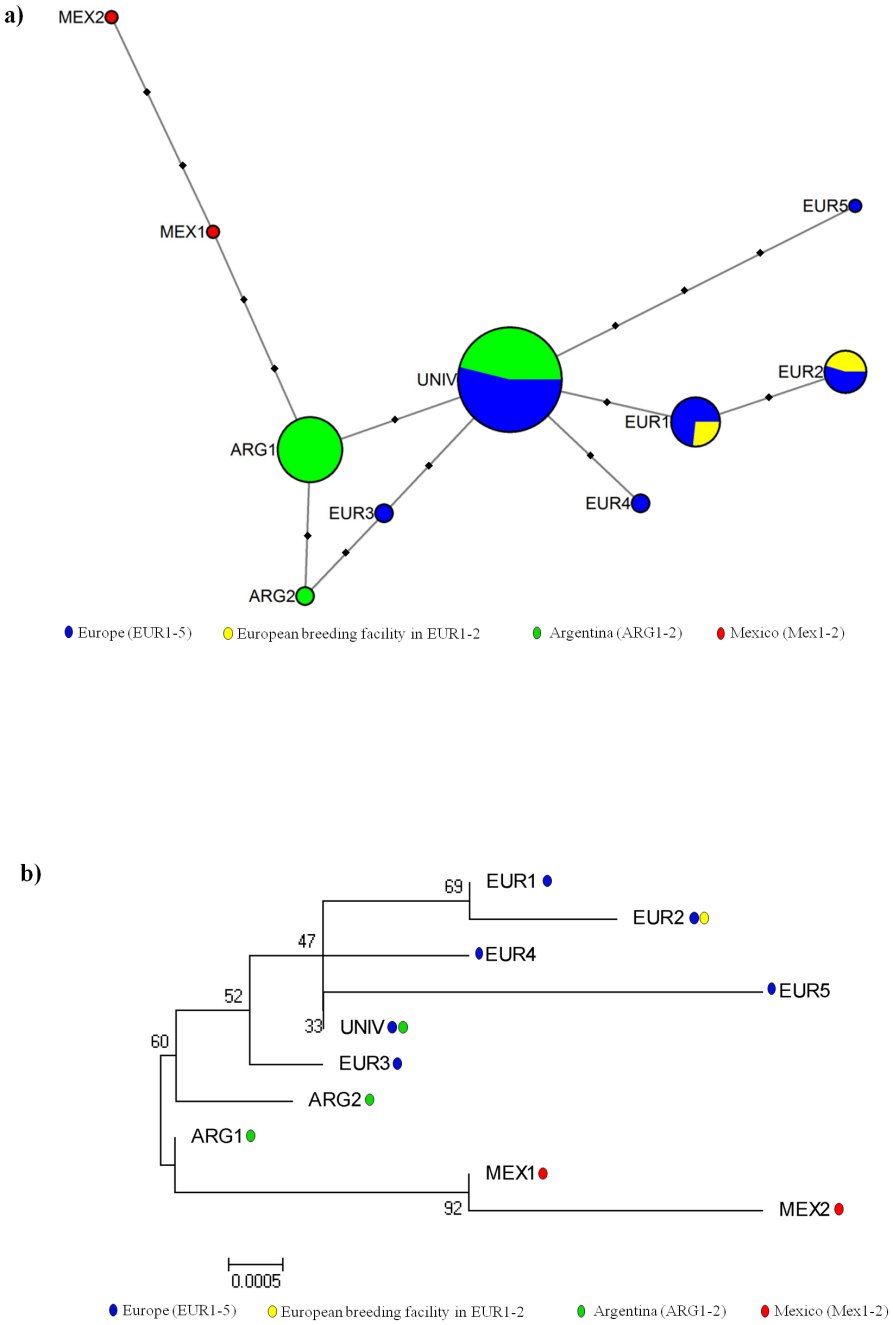
Network and evolutionary relationships of *Apicystis bombi* haplotypes. (a) Bees in Europe (blue), Argentina (green), Mexico (red), and a commercially produced bumblebee colony in Europe (yellow). Circle sizes are proportional to the numbers of bees infected with the haplotype and the circle colors indicate the proportion of bees with the haplotype that came from each location. The black squares represent unobserved single-nucleotide substitutions. (b) The geographic location in which each haplotype was detected is indicated with colored spots for Europe (blue), Argentina (green), Mexico (red), and a commercially produced bumblebee colony in Europe (yellow). The optimal tree with the sum of branch length = 0.0176 is shown. The percentages of replicate trees in which the associated haplotypes clustered together in the bootstrap test (1000 replicates) are shown next to the branches.

We classified *A. bombi* haplotypes according to their geographic location, and also included an infected *B. terrestris* sample from a European commercially produced bumblebee colony (see [15]). We found three haplotypes in Argentina (ARG1, ARG2 and UNI, in nine hosts), six haplotypes in Europe (EUR1–EUR5 and UNI, in eight hosts), two haplotypes in Mexico (MEX1 and MEX2, in a single host), and two haplotypes in a commercially produced colony (EUR1 and EUR2). Haplotypes EUR1, EUR2, EUR3, EUR4 and EUR5 were only found in European samples, ARG1 and ARG2 were only found in Argentine samples, and the haplotypes MEX1 and MEX2 were only found in Mexico (**Figure 3a**). The universal haplotype UNI was detected in all Argentine samples, and in 75% of the European samples. The other prevalent haplotype ARG1 occurred in 78% of the samples in Argentina while it remained undetected in our sampling in Europe. Furthermore, as shown in **figure 3b**, the haplotypes found in Europe and Argentina formed a single phylogenetic clade. The genetic variation among geographic regions (Argentina and Europe) only explained 15.2% of the total genetic variation, with most of the variation found among and within specimens of each region (43% and 42%, respectively) (**Table 1c**). The genetic structure between Europe and Argentina was therefore small and there was no significant phylogenetic structure across these regions (**Figure** **3b**). The two haplotypes found within the commercially produced bumblebee colony also fell within this European/Argentine cluster.

### Genetic Diversity of *A. bombi* in Argentina and Europe

The mean sequence divergence of *A. bombi* among specimens from Argentina (mean ± s.d. = 0.7±0.6, n = 9) was significantly smaller than the mean sequence divergence among European specimens (1.5±1.3, n = 8; Mann Whitney U test: z = −4.98; *P*<0.001). Indeed, only three haplotypes were found among Argentine specimens separated by one or two mutations, while the European haplotypes were more diverse (**Figure 3**).

## Discussion

### The ITS Region can be used to Haplotype *A. bombi*

In this study we used ITS1 and ITS2 as a molecular marker to study genetic variability of *A. bombi*. Although these markers have been widely used, particularly because of their high level of sequence variation, there are caveats regarding their use [31]. Parasites have multiple rDNA loci which makes intragenomic diversity possible, as for example reported for the bumblebee parasite *Nosema bombi* [32]. Because of concerted evolution, intragenomic diversity is generally low [31]. However, different paralogs are possible, making ITS markers uninformative if intragenomic diversity is higher than the intergenomic diversity within a population. In our data, 34% of the variability was within individuals. This percentage may reflect intragenomic diversity or infection with multiple strains. Although we cannot differentiate between these two explanations, we speculate that there is little intragenomic diversity for *A. bombi* parasites for two reasons. First, 16 out of 18 specimens contained either one (7out of 16 specimens) or two (9 out of 16 specimens) haplotypes (across 10 clones). Second, it is often reported that multiple parasite strains infect a single host [33], a phenomenon reported for other bumblebee parasites like *Crithidia bombi* [34], [35].

### Honeybees and Bumblebees Share *A. bombi* Haplotypes

Our results indicate that honeybees and the two European bumblebee species, *B. terrestris* and *B. ruderatus,* sampled from the same area in Argentina, were all infected by the same haplotypes of *A. bombi*. This parasite was originally described as a bumblebee parasite, implicated in increased mortality rates in infected queens (*B. pratorum*; [22]). These authors found that the parasite re-emerges in bumblebee workers late in the season, suggesting that some queens survive infection and transmit the parasite to the progeny and/or alternate reservoirs exist for the parasite [22].

Recent studies suggest that *Ap. mellifera* could represent an alternative reservoir and/or vector of *A. bombi* [13], [23], [24,]. Although we have demonstrated that the same parasite species -and haplotypes within the species- is present in both taxa, we cannot conclude whether *A. bombi* found in honeybees can indeed re-infect bumblebees. Furthermore, it still needs to be determined whether the *A. bombi* found in Argentina represented genuine infections of *Ap. mellifera*. The fact that honeybee and bumblebee populations are largely sympatric throughout the geographic range of *Bombus*, which is a first requirement for parasites to jump between host species [36], makes the presence of shared pathogens probable. Indeed, several honeybee parasites are now known to infect bumblebees [13], [37], [38], [39], [40], [41], showing that species jumps between bumblebees and honeybees do not necessarily result in a dead-end host. Furthermore, recent work has confirmed that *A. bombi* from bumblebees can readily infect honeybees [13]. Although knowledge about multi-host parasites is ‘scarce’, the high rate of interspecies transmission plays a central role in the evolution of virulence in host-parasite networks [42].

### The Same *A. bombi* Haplotypes are Shared between Argentina and Europe

Our fundamental question was whether *A. bombi* detected in Argentina is indigenous or from introduced bees originating from Europe. If *A. bombi* were indigenous in two geographically separated continents, two clearly differentiated clusters should be seen, where one contains parasites of Argentina and the other of Europe. Two separate clusters would also imply that the introduced European bees became infected with *A. bombi* already present in Argentina. Instead we found that the European parasites clustered together with those from Argentina. However, we note that the detection of only one cluster could be the consequence of insufficient sampling in Argentina and/or Europe. Higher sample numbers could reveal separating clusters. However, the homogeneity in the data between Europe and South America strongly suggests that parasites identified in the analyzed samples share the same origin.

We detected low geographic structuring between Europe and Argentina, with only 15.2% of the genetic variation being explained by location. Indeed both locations share the most frequent haplotype (UNI). The haplotypes detected in Argentina (ARG1 and ARG2) are one and two point mutations different from UNI, respectively (**Figure 3a**). This actually makes them indistinguishable from most of the haplotypes (EUR1, EUR2, EUR3 and EUR4) exclusively found in Europe. Thus the differences detected between Europe and Argentina are of the same magnitude as the those found within European samples. Geographical structuring in parasites between land masses separated by an ocean has been observed in other taxa; a good example is *Atractolytocestus huronensis* (Cestoda: Caryophyllidea), an invasive parasite of common carp introduced from North America to Europe, which exhibits at least 8 mutations or 1.2% sequence diversity in ITS2 of the two geographic locations [43]. We recognize here that one has to be careful when comparing mutational rates of the ITS region of different species. Ideally, reference samples of *A. bombi* from isolated locations without non-native bumblebees or honeybees would be required to quantify the natural genetic structuring of *A. bombi* within its natural habitats. With the intense and worldwide transport of honeybees and bumblebees such samples are very difficult to obtain. However, it is evident from our data that the weak structuring of *A. bombi* between European and Argentine bumblebees does not reflect the historical Palearctic, Nearctic, and Neotropic separation of the Old World *Bombus* ancestor [44]. Rather, it supports a common and more recent origin, which is consistent with the hypothesis that introduced European bees (be it *Apis* and/or *Bombus*) carried *Apicystis* into Argentina.

The *A. bombi* haplotypes found in *B. ephippiatus*, native to Mexico, are more distantly related, demonstrating that the ITS region of *A. bombi* is variable enough to identify differentiation at these spatial scales where present. These data also represent the first record of *A. bombi* in this country. *A. bombi* was originally described as *Mattesia bombi* [45], [19] and has been found in Ontario (Canada) [45], suggesting its presence in North America before bumblebee transport started. However, the single sample from Mexico is insufficient to draw any conclusion regarding genetic differentiation within Mexico. The native *B. ephippiatus* is found in a region where non-native commercially produced bumblebees, *Bombus impatiens*, have become established [46], [47]. It is uncertain whether the *A. bombi* haplotypes in the Mexican bee represent indigenous, North American haplotypes, or haplotypes spilling over into Mexico from other locations.

The Mexico sample does further emphasize the need for a worldwide prevalence and haplotyping study in which regions with intense bumblebee and/or honeybee importation are compared with regions without importation.

### Geographic Transmission Routes of *A. bombi* and Perspectives

Our molecular haplotyping strongly suggests that the origin of the *A. bombi* currently found in non-native Argentine bees is the same as those found in Europe. What mechanisms could explain this? A first possibility is that *A. bombi* originated from Europe and was subsequently introduced into Argentina either by *Ap. mellifera*, *B. terrestris* or *B. ruderatus*. We have one infected bumblebee sample originating from a commercially-produced bumblebee colony, and its *A. bombi* haplotypes fall within the European/Argentine cluster. This again indicates there is a shared origin of *A. bombi*, but remains uninformative about the original native region of *A. bombi*. Furthermore, the original native locality of commercially produced bumblebees is not always clear, and thus also of the parasites they carry. The two subspecies most commonly used in European breeding factories are *B. t. terrestris* and *B. t. dalmatinus*, with the former originating from a variety of European countries and the latter from Greece and Turkey [48]. In addition, Israeli facilities also supplied bumblebees to Chile. Together with the fact that *A. bombi* has been detected in Turkey [49], this makes the Mediterranean region a good candidate to retrieve the original location of the ARG1, ARG2 haplotypes.

A second possibility is that *Ap. mellifera* became infected with *A. bombi* from a South American host and global honeybee queen transport subsequently homogenized the genetic variation of this parasite. Although this seems a less probable explanation, it is a possibility that the current results cannot exclude. At a minimum, however, *A. bombi* can be considered as an emergent disease in Argentina, in either the native or non-native bees depending on the direction of transfer, with the implication that it may have also rapidly increased in prevalence in other regions where non-native honeybees and bumblebees have been introduced. It has been recognized that emergent diseases of wild populations pose a substantial threat to the conservation of global biodiversity [14], [50]. With bee diversity already being threatened by multiple factors [3], monitoring of this emergent infectious disease is crucial. Here we present molecular tools to study intra- and inter-species diversity to untangle the spread of this parasite. In order to detect the exact origin and transmission dynamics of this parasite, multiple locations now need to be sampled worldwide, especially the Mediterranean region, preferably including regions with no honeybee and/or bumblebee imports. In parallel, pathological studies are also urgently needed to assess the virulence of the parasite across native and non-native hosts.
